# Why dogs prefer zoomies to zoom and what it tells us about the importance of in-person meetings for learning and memory

**DOI:** 10.1007/s10339-024-01235-8

**Published:** 2024-10-14

**Authors:** Géraldine Coppin, Michael L. Onofrio

**Affiliations:** 1 Department of Psychology, UniDistance Suisse, Schinerstrasse 18, 3900 Brigue, Switzerland; 2 Swiss Center for Affective Sciences, University of Geneva, Geneva, Switzerland; 3 Tillster, Inc., 5959 Cornerstone Court West #100, San Diego, CA 92121 USA

**Keywords:** In-person meetings, Online meetings, Body odors, Learning, Chemosensory communication

## Abstract

As people commonly observe dog behaviors like the sudden bursts of physical movement colloquially known as “zoomies,” and the canine penchant for sticking their nose out of car windows and for sniffing intently in dog parks, it is not surprising that people generally believe dogs learn and communicate by smell. While people generally discount their own olfactory sensitivity and the importance of smell overall, humans also learn and communicate by smell, in some cases even better than dogs. In this article, we discuss why this information exchange matters for learning and memory and why virtual meetings don’t pass the sniff test.

## Main text

A considerable amount of research has been conducted and is ongoing related to the effects of the covid-19 virus on the senses of smell and taste (e.g., Liu et al. 2022). For instance, the Global Consortium for Chemosensory Research (https://gcchemosensr.org/) has been working on the COVID-19’s effects on smell and taste since the beginning of the pandemic. Both traditional and social media have portrayed stories of acute and long-lasting cases of individuals with distortions and/or losses of smell and taste. However, a more indirect effect of the covid-19 pandemic on our olfactory experience is often overlooked: the shift to online meetings and conferences has deprived us from smelling each other and making the most of our social interactions.

There are plenty of reasons to be in favor of going back to in-person meetings and conferences (e.g., “zoom fatigue” and increased creativity; see Brucks and Levav 2022) or to be opposed to it (e.g., ecological concerns when travel is required). We do not intend to settle this debate. Here, we will only point out why attending events in person might be valuable for learning and memory from an olfactory perspective: being in person rather than online enables chemosensory communication and facilitates the acquisition and retention of valuable information.

People tend to doubt their sense of smell more than other senses. This may be because odor perception is often implicit (Köster et al. 2014). Yet, humans have excellent olfactory abilities (McGann 2017). Humans can detect a very large range of odors, may be more sensitive than rodents or dogs to certain odors such as wine (McGann 2017) and can be trained to track scents (Porter et al. 2007). While we often discount the importance of chemosensory communication, humans tend to smell various parts of their own bodies, clothing, offspring, social and professional acquaintances and romantic acquaintances (Perl et al. 2020). In social settings, we tend to sample others’ body odors repeatedly, especially after shaking someone’s hand (Frumin et al. 2015).

Why do we do this? Human body odors are complex chemical mixtures of numerous compounds that can convey information we are only beginning to discover. The effects of body odors on cognitive and affective processes tend to occur below conscious levels (e.g., Parma et al. 2017). We know body odors can inform us about the other person’s age (Mitro et al. 2012), gender (Doty et al. 1982), diet (Havlicek and Lenochova 2006), hygiene (Penn and Potts 1998), sickness (Olsson et al. 2014), hormonal levels (Rantala et al. 2006), genetic proximity (Winternitz et al. 2017), emotional states (Groot and Smeets 2017), personality traits (Sorokowska et al. 2012) and even their potential for social bonding (Ravreby et al. 2022). In a professional context such as a meeting or a conference, one may expect the ability to detect this information as immensely valuable. This is true for all researchers but may be especially impactful for those who started attending meetings during the pandemic and have not yet met many of their colleagues in person (e.g., O’Brien 2021).

Olfaction is unique in terms of emotional and memory encoding (e.g., Delplanque et al. 2017). Unlike all the other sensory modalities, olfactory information does not go through the thalamus before reaching the primary sensory cortex (e.g., Price and Powell 1971). Memories evoked by olfactory cues are more emotional and evocative than memories evoked by visual or auditory cues (e.g., Herz et al. 2004). The unique power of odors to evoke vivid memories from the past even has a name—the “Proust effect” (e.g., Chu and Downes 2002). Moreover, first olfactory associations have a privileged brain representation, i.e. early olfactory associations have a unique hippocampal activation, which is not the case for early auditory associations (Yeshurun et al. 2009). Finally, there is a large overlap in brain structures responsible for olfactory, affective and memory processing (e.g., Soudry et al. 2011).

In the specific case of body odors, we know that learning about someone else from chemosensory information has some underestimated social benefits (e.g., Groot et al. 2017; Sullivan et al. 2015). We can recognize faces better when a body odor is presented at the same time (Cecchetto et al. 2019), even the body odor is masked. Thus, body odors can act as contextual cues increasing memory for faces. This effect is significantly higher than the one found with common odors. In other words, associating a face on a zoom meeting with an everyday odor will not promote memory for this face as much as actually being in the same room with this person, where his/her body odor is present. Besides body odors, odors such as perfumes can also communicate social messages (Groot et al. 2017). Integrating social information from all sensory modalities, including chemosensory information, seems to consequently be essential to social learning and memory. This is true for learning about both enduring characteristics (e.g., sex) and dynamic states (e.g., emotions).

When looking at individuals suffering from anosmia, i.e., who have lost their sense of smell, one can see their close social relationships are negatively impacted (Blomkvist and Hofer 2021). New social relationships would be particularly affected, and we can speculate it might partly because of the inability to learn from the other through chemosensory communication. The impact on their professional relationships is rarely studied systematically, but such research could provide valuable insights into what is lost by meeting online. Close relationships might be of concern to researchers too: it is common for scientists, and academics in general, to be romantically involved with others in their field. For instance, 36% of faculty members in the United States were part of an academic couple according to a 2008 report (Clayman 2008). Whether for initiating or maintaining a romantic relationship, the processing of body odors likely plays a key role (Mahmut and Croy 2019) and has undoubtably suffered as conference liaising has become a virtual affair.

Some conference organizers strongly recommend or even require masking as a condition for attendance. The ability to detect and process chemosensory information will likely change if the event requires the wearing of a mask, though data here is lacking. A study conducted on a modest sample size (N = 20) suggested that wearing a surgical mask did not significantly affect odor identification, though it reduced odor sensitivity and odor intensity ratings (Chen et al. 2020). The reduced effect on odor sensitivity was recently replicated in healthcare workers (Xia et al. 2023). We do not know yet how this reduced odor sensitivity may affect learning and memory from chemosensory information in a social setting.

Besides wearing a mask, other factors can impact the effect of odors. The effect of concentration and spatio-temporal distribution of odors are still not perfectly understood (e.g., Pannunzi and Nowotny 2019) but the impact of an odor will be influenced by the methods of odorant delivery (e.g., jars, sniffin’ sticks, olfactometer, see for instance Johnson et al. 2006), the concentration of the odorant, the surrounding environment or the number of individuals present in a room. While large settings like conference halls may dilute chemosensory information, these environments frequently involve the simultaneous use of multiple senses. As conferences typically facilitate and encourage interpersonal interaction, it is plausible that multi-modal cues help attendees identify others for subsequent proximate interpersonal interaction, where more concentrated olfactory information exchange could occur. The notion of seeking physically proximate interactions to gather additional olfactory information has been observed in animals (Marin et al. 2021). In humans, cues from different sensory modalities help us quickly categorize visual inputs as well, and olfaction may play a key role in clarifying ambiguous visual information (see e.g., Rekow et al. 2022).

## Conclusion

Until we gain a deeper understanding of the breadth of information transmitted through smell and develop an effective chemosensory closed captioning system, virtual meetings will not provide as much information as in-person meetings. Until then, we would like to remind you that in-person attendance at your next conference may increase your chances of finding, learning about, and remembering not only your next collaborator but possibly even your next cohabitator.
